# Changes in a Protein Profile Can Account for the Altered Phenotype of the Yeast *Saccharomyces cerevisiae* Mutant Lacking the Copper-Zinc Superoxide Dismutase

**DOI:** 10.3390/metabo13030459

**Published:** 2023-03-22

**Authors:** Magdalena Kwolek-Mirek, Aleksandra Dubicka-Lisowska, Sabina Bednarska, Renata Zadrag-Tecza, Pawel Kaszycki

**Affiliations:** 1 Department of Biology, Institute of Biology and Biotechnology, College of Natural Sciences, University of Rzeszow, 35-601 Rzeszow, Poland; sbednarska@ur.edu.pl; 2 Department of Plant Biology and Biotechnology, Faculty of Biotechnology and Horticulture, University of Agriculture in Krakow, 31-425 Krakow, Polandpawel.kaszycki@urk.edu.pl (P.K.)

**Keywords:** copper-zinc superoxide dismutase, methionine auxotrophy, oxidative stress, superoxide anion, yeast, proteome mapping, two-dimensional gel electrophoresis, mass spectrometry

## Abstract

Copper-zinc superoxide dismutase (SOD1) is an antioxidant enzyme that catalyzes the disproportionation of superoxide anion to hydrogen peroxide and molecular oxygen (dioxygen). The yeast *Saccharomyces cerevisiae* lacking *SOD1* (Δ*sod1*) is hypersensitive to the superoxide anion and displays a number of oxidative stress-related alterations in its phenotype. We compared proteomes of the wild-type strain and the Δ*sod1* mutant employing two-dimensional gel electrophoresis and detected eighteen spots representing differentially expressed proteins, of which fourteen were downregulated and four upregulated. Mass spectrometry-based identification enabled the division of these proteins into functional classes related to carbon metabolism, amino acid and protein biosynthesis, nucleotide biosynthesis, and metabolism, as well as antioxidant processes. Detailed analysis of the proteomic data made it possible to account for several important morphological, biochemical, and physiological changes earlier observed for the *SOD1* mutation. An example may be the proposed additional explanation for methionine auxotrophy. It is concluded that protein comparative profiling of the Δ*sod1* yeast may serve as an efficient tool in the elucidation of the mutation-based systemic alterations in the resultant *S. cerevisiae* phenotype.

## 1. Introduction

Superoxide dismutase (SOD, EC 1.15.1.1) is an antioxidant enzyme that catalyzes the two-step disproportionation of superoxide anion to hydrogen peroxide and molecular oxygen (dioxygen). In the yeast *Saccharomyces cerevisiae,* there are two SOD isoenzymes—a copper- and zinc-containing enzyme (CuZnSOD, SOD1) occurring in the cytosol and in the intermembrane space of mitochondria, and a manganese-containing enzyme (MnSOD, SOD2) localized in the mitochondrial matrix [1,2]. The Sod2p protein and the mitochondrial fraction of Sod1p are involved in the disproportionation of superoxide anion generated by the mitochondrial respiratory chain [2,3,4], while the cytosolic fraction of Sod1p is mainly utilized for peroxide-mediated signaling [5,6]. Sod1-derived hydrogen peroxide stabilizes a pair of plasma membrane casein kinases, Yck1p and Yck2p, that control nutrient sensing and energy metabolism [7,8,9], and plays a role in antioxidant defense by stimulating the production of NADPH [10]. Sod1p also participates in controlling the level and activity of the voltage-dependent anion-selective channel (VDAC) responsible for the proper transport of metabolites between the intermembrane space of mitochondria and cytosol [11,12]. Furthermore, in response to elevated endogenous and exogenous reactive oxygen species (ROS), Sod1p may rapidly relocate into the nucleus, then bind to the promoters and regulate the expression of genes involved in antioxidant defense and DNA damage repair [13].

The yeast *S. cerevisiae* seems to be an ideal model for studying the biological role of CuZnSOD. SOD1 accounts for approximately 80–90% of the total superoxide dismutase activity during yeast growth in media supplemented with glucose as a source of carbon [14]. The yeast that lacks *SOD1* (Δ*sod1* mutant) displays a number of oxidative stress-related alterations in its phenotype as compared to the wild-type strain. These include a lower growth rate in air, inability to grow in an atmosphere of 100% oxygen in a rich cultivation medium, lysine and methionine auxotrophies [15], elevated free iron concentration [16], and inactivation of proteins containing the 4Fe-4S groups [17]. Moreover, the Δ*sod1* mutant exhibits an increased sensitivity to oxidative stress-inducing agents that either generate ROS, such as paraquat and menadione [18,19], or decrease the level of reduced glutathione (GSH) e.g., dithiopyridine [20] and acrolein [21]. The depletion of *SOD1* was also shown to increase intracellular ROS content [22,23], promote protein carbonylation [24], and [*PSI*^+^] prion formation [25], as well as result in fragmentation of both nuclear DNA [13,26] and vacuoles [27]. In addition to the above, the *SOD1* mutation was reported to stimulate the production of the cell wall chitin and to elevate sensitivity to the cell wall-perturbing agents [28]. Furthermore, the loss of either *SOD1* alone or both *SOD1* and *SOD2* dramatically reduced the chronological and replicative lifespans of the yeast [29,30,31,32]. Interestingly, the depletion of *SOD1* increased cellular GSH content [22,23], apparently as a compensatory response.

The research on the involvement and importance of superoxide dismutase in the oxidative stress protection of cells has been conducted for many years, though still many issues require better understanding. We decided to search for an explanation of the pleiotropic Δ*sod1* mutant phenotype in the yeast *Saccharomyces cerevisiae* at the proteomic level. We focused on a comparative protein electrophoretic profiling of the wild-type strain and the *SOD1*-lacking mutant, followed by mass-spectrometry-based identification of differentially expressed proteins. Such an approach enabled us to define the main functional protein categories responsible for the altered mutant yeast phenotype. To our best knowledge, no other study attempted to elucidate the mechanisms of complex pleiotropic effects of the Δ*sod1* mutation on the basis of comparative proteomics.

## 2. Materials and Methods

### 2.1. Yeast Strains and Growth Conditions

The following yeast strains were used: wild-type SP4 MATα *leu1 arg4* [33], and Δ*sod1* mutant, isogenic to SP4, MATα *leu1 arg4 sod1::natMX* [34]. The yeast was grown in the standard liquid YPD medium (1% Yeast Extract, 1% Yeast Bacto-Peptone, 2% glucose) on a rotary shaker at 150 rpm, at the temperature of 28 °C.

### 2.2. Protein Extraction

The cells from the exponential phase of culture (~16 h) were centrifuged (4000× *g*, 4 min, 4 °C), washed twice with MilliQ water, and suspended in a cold homogenization buffer (20 mM phosphate buffer, pH 6.8, containing 1 mM EDTA, 0.2% DTT, and 1 mM PMSF). Then, the biomass was disrupted with 0.5 mm glass beads in 6 cycles of 30 s with intervals for cooling the sample on ice and then centrifuged (14,000× *g*, 15 min, 4 °C). Supernatants were transferred to fresh tubes and immediately frozen at −80 °C. Four independent biological replicates were prepared for each strain. Protein concentration was determined using the Bradford method.

### 2.3. Two-Dimensional Gel Electrophoresis (2-DE)

The protein samples were separated by two-dimensional gel electrophoresis. In the isoelectrofocusing step (IEF, the first dimension), the whole-cell protein extracts were loaded onto 7 cm IPG strips (Bio-Rad, Hercules, CA, USA) with pI ranging from 3 to 10. A sample of 50 µg of the protein extract was dissolved in a rehydration buffer (7 M urea, 2 M thiourea, 2% CHAPS, 0.002% bromophenol blue, 20 mM DTT, and 1% ampholyte buffer BioLyte (Bio-Rad)) to a final volume of 150 µL, and then applied onto an IPG strip. Strips were rehydrated passively for 12 h at 20 °C, followed by an isoelectrofocusing run using the Protean IEF Cell (first step: 250 V for 20 min, second step: 4000 V for 120 min, third step: 4000 V, 10,000 Volt-hours) at 20 °C with a current limit of 50 µA per strip. Prior to the SDS-PAGE, the IPG strips were equilibrated for 10 min in buffer I (1% DTT, 6 M urea, 75 mM Tris HCl, pH 8.8, 30% glycerol, 2% SDS) and then for 10 min in buffer II (2.5% iodoacetamide, 6 M urea, 75 mM Tris HCl, pH 8.8, 30% glycerol, 2% SDS). The SDS-PAGE step (the second dimension) was carried out according to Laemmli [35] using Protean II xi Cell 16 × 16 cm slab unit (Bio-Rad), using 4% stacking and 10% separating polyacrylamide gels. In order to maximize the reproducibility of the spot patterns and optimize the matching of the protein profiles, both IPG strips, obtained upon IEF of the wild-type strain and the Δ*sod1* mutant were placed next to each other onto one SDS-polyacrylamide gel (the “two-in-one gel” technique) and then overlaid with low melting point agarose (ReadyPrep overlay agarose, Bio-Rad). Protein electrophoretic separation was performed at 20 mA per gel, typically for 6 h. Proteins were detected with silver staining according to Jungblut and Seifert [36]. Note that three protein extracts (both WT and Δ*sod1*) obtained upon three independent physiological experiments were used for proteome mapping and matching. To enable efficient MS analysis, additional, independent electrophoresis was carried out followed by gel staining without glutaraldehyde.

### 2.4. Image Analysis and Statistics

The resultant 2-DE proteome maps were digitalized and matched to identify differentiating spots. The 2-DE gel image analyses were performed using AzureSpot Analysis Software (Azure Biosystems, Dublin, CA, USA). Spot detection and matching were manually revised in the software. Protein spot intensities were normalized with regard to the total density of the gel images. For all the differentiating spots, changes in protein abundances were evaluated quantitatively based on the analysis of the respective spot intensities as determined for the Δ*sod1* mutant and the WT strain. In order to obtain the most reliable and repetitive data, only these spots were selected for further analyses, which had been identified as differentiating ones in all of the electrophoretic runs. Quantitative variations were statistically validated using a Student’s t-test and the statistical analysis was performed employing the SPSS 21.0 software.

### 2.5. Mass Spectrometry

The protein spots were excised from the gel and analysed by liquid chromatography coupled to the mass spectrometer in the Laboratory of Mass Spectrometry, Institute of Biochemistry and Biophysics, Polish Academy of Sciences (Warsaw, Poland). Samples were subjected to a standard procedure of trypsin digestion during which proteins were reduced with 100 mM DTT (30 min at 56 °C), alkylated with 0.5 M iodoacetamide (45 min in a darkroom at room temperature), and digested overnight with 10 ng/µL trypsin (Promega, Madison, WI, USA) at 37 °C. The peptide mixtures were concentrated and desalted on an RP-C18 precolumn (Waters, Budapest, Hungary), and further peptide separation was achieved on a nano-Ultra Performance Liquid Chromatography (UPLC) RP-C18 column (Waters, BEH130 C18 column, 75 µm i.d., 250 mm length) of a nanoACQUITY UPLC system, using a 160 min gradient from 5 to 30% of acetonitrile. The column outlet was directly coupled to the electrospray ionization (ESI) ion source of the Orbitrap Elite type mass spectrometer (Thermo Scientific, Waltham, MA, USA), working in the regime of data-dependent MS to MS/MS switch with HCD type peptide fragmentation. An electrospray voltage of 2 kV was used. A blank run to ensure there was no cross contamination from previous samples preceded each analysis.

### 2.6. Protein Identification

Raw data files were preprocessed with Mascot Distiller software (ver. 2.4.2.0, MatrixScience). The obtained peptide masses and fragmentation spectra were matched to the *Saccharomyces* Genome Database (SGD; 9387 sequences/4,227,730 residues) using the Mascot search engine (Mascot Daemon ver. 2.4.0, Mascot Server ver. 2.4.1, MatrixScience). The search parameters were as follows: enzyme specificity—trypsin; the protein mass was left as unrestricted; mass values as monoisotopic with one missed cleavage being allowed; peptide mass tolerance ±30 ppm; fragment mass tolerance ±0.1 Da. Alkylation of cysteine by carbamidomethylation was set as fixed and oxidation of methionine was a variable modification.

### 2.7. Western Blot

The protein samples were separated by SDS-PAGE and then transferred to nitrocellulose membrane (PVDF Western Blotting Membranes, Roche) by semidry immunoblotting (Bio-Rad). The presence of proteins on the membrane was confirmed by Ponceau S (Sigma-Aldrich, St. Louis, MI, USA) labeling. After blocking with PBST buffer (PBS, 0.1% Tween 20) containing 3% nonfat milk, the following primary antibodies were used: the antiyeast methionine synthase (MET6; 1:500, X-P05694-N, Abmart, Berkeley Heights, NJ, USA) and the antiyeast alcohol dehydrogenase (1:5000, ab34680, Abcam). The respective proteins were detected after incubation with the horseradish peroxidase-conjugated secondary antibodies (1:10,000, AP160P, Millipore, Merck, and 1:10,000, 111,035,003, Jackson ImmunoResearch, respectively) with a SuperSignal West PICO Chemiluminescent Substrate (Pierce Biotechnology, Waltham, MA, USA), according to the manufacturer’s protocol. The images were captured using an Azure c300 Imaging Systems.

### 2.8. RNA Samples

The RNA samples were obtained using a GeneMATRIX Universal RNA Purification Kit according to the manufacturer’s protocol (EURx, Gdansk, Poland). Cells from the exponential phase of culture (5 × 10^7^ cells/mL) were centrifuged, washed twice with MilliQ water, and suspended in the spheroplast buffer (1 M sorbitol, 0.1 M EDTA, 0.1% β-mercaptoethanol) containing lyticase (250 U per sample) for 30 min at 30 °C. The resultant spheroplasts were used for RNA isolation. The RNA samples were stored at −20 °C and each of them was thawed only once. The concentration and purity of RNA samples were measured with a Tecan Infinite M200 reader (Tecan Group Ltd., Männedorf, Switzerland) equipped with a NanoQuant Plate using a 260 nm/280 nm ratio.

### 2.9. Real-Time PCR

For first strand cDNA synthesis, 1 µg of RNA was taken using SuperScript IV VILO Master Mix with ezDNase enzyme (Thermo Fisher Scientific, Waltham, MA, USA) according to the manufacturer’s protocol, and the samples were stored at −20 °C until use. Real-time PCR was performed using LightCycler 96 (Roche Life Science, Penzberg, Germany) equipment and TaqMan chemistry. Briefly, the cDNA sample was diluted, mixed with TaqMan Fast Advanced Master Mix and TaqMan Gene Expression Assays (Applied Biosystems, Waltham, MA, USA, Life Technologies, Carlsbad, CA, USA), and PCR reaction was run in five independent repetitions. The *MET6* gene expression was tested and the *ACT1* gene was used as an internal control gene. The relative gene expression was calculated with the −ΔΔC_T_ method for comparison of the individual gene expression between the WT strain and Δ*sod1* mutant.

## 3. Results

### 3.1. Comparative Proteome Analysis

**Figure 1:**
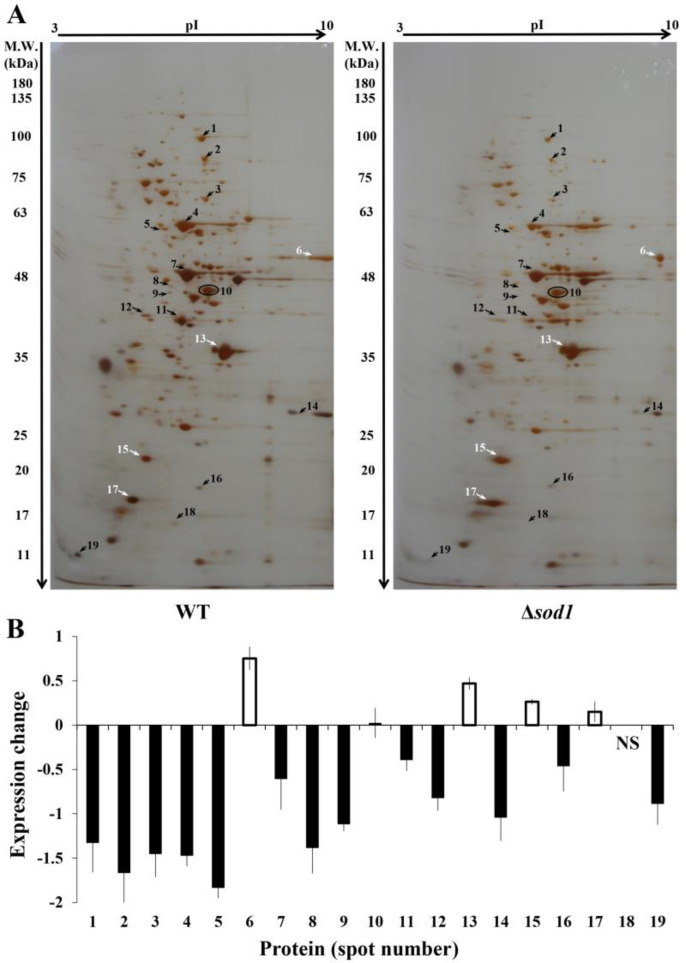
Comparison of the WT strain and Δ*sod1* mutant proteomes (**A**) Representative two-dimensional gel electrophoresis of protein extracts of the WT strain (lefthand side of the gel) and the Δ*sod1* mutant (righthand side of the gel). Differentially expressed proteins are indicated in the gels with respective numbers. Downregulated proteins are indicated by black arrows and upregulated ones by white arrows. Protein spot number 10 is a control (indicated by a circle). (**B**) Expression change (Log2 of fold) of proteins was calculated for the respective protein 2-DE spots of the Δ*sod1* mutant and the WT strain. Data are presented as a mean ± SD of three independent experiments. NS—no protein spot for the Δ*sod1* mutant.

In order to compare proteomes of the wild-type strain and the Δ*sod1* mutant, we performed a 2-DE analysis of the whole-cell protein extracts. For the first dimension, wide-range IPG strips were used (pI range 3–10) to enable the detection of most of the proteins involved in cellular metabolism. Eighteen electrophoretic spots with differential protein expression were detected repetitively in three independent experiments (Figure 1 and Figure S1). Fourteen of the proteins were downregulated (spots numbered 1–5, 7–9, 11, 12, 14, 16, 18, and 19 of Figure 1A, all indicated by black arrows) and four were upregulated (spots numbered 6, 13, 15 and 17 of Figure 1A, white arrows). The abundances of individual proteins were quantified by comparison of intensities of the respective spots for the Δ*sod1* mutant and the WT strain and were presented as the value of the expression change parameter (given as Log2 of fold) (Figure 1B). As a control, one protein (spot number 10 of Figure 1A, indicated by a circle) was chosen for further analysis, whose expression was unchanged for both strains (*cf.* Figure 1A,B; [37]).

### 3.2. Identification of Proteins

Nineteen proteins were identified with high confidence based on the scores and sequence coverage from the *Saccharomyces* Genome Database (SGD) (Table 1). The most abundant protein in the studied yeast extracts was ENO2 (spot number seven of Figure 1A) for which the exponentially modified Protein Abundance Index (emPAI) was 43.84 (Table 1). On the other hand, among the proteins with the least expression were TEF1/TEF2 and DYS1 (spots numbers six and eight of Figure 1A), whose emPAI were calculated as 1.01 and 1.09, respectively (Table 1).

In the case of three electrophoretic spots (numbers one, three, and six of Figure 1A), more than one protein was attributed to each particular spot. These proteins were present in the form of paralogs (spots numbered one, three and six, that is: EFT1 and EFT2; ADE17 and ADE16 as well as TEF1 and TEF2, respectively) (Table 1). Paralogous genes (proteins) are created by a duplication event and they often have a similar or the same function in the cell, though sometimes a duplicated gene may acquire new functions through mutation [38,39].

Importantly, the SOD1 protein was present, as expected, only in the WT strain and no respective spot was observed for the Δ*sod1* mutant (protein spot number 18 of Figure 1A). The above result proves the validity of our experimental model employed for the study. It should be pointed out here that the use of yeast strain, which was obtained by disruption of the *SOD1* gene [34] allows for the assumption that all the phenotypic alterations as observed for the Δ*sod1* mutant are indeed effects of the lack of the single superoxide dismutase one gene and, consequently, its protein product.

### 3.3. Functional Classes of the Identified Proteins

**Figure 2:**
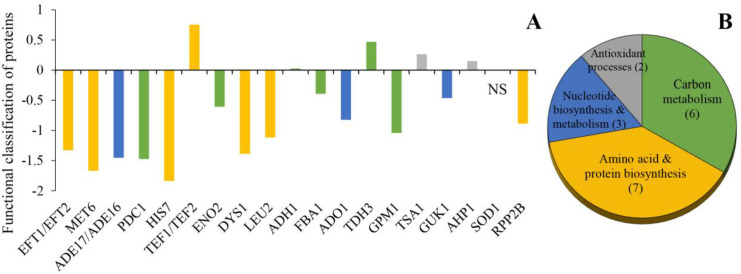
Functional classification of proteins with altered expression in the Δ*sod1* mutant. (**A**) Identified proteins are divided into four functional categories: carbon metabolism—marked in green, amino acid and protein biosynthesis—marked in yellow, nucleotide biosynthesis and metabolism—marked in blue and antioxidant processes—marked in violet. Values above and below zero on the graph indicate protein up- and downregulated, respectively. NS—no protein spot for the Δ*sod1* mutant. (**B**) Number of identified proteins in each category and the proportion between them are shown in the pie chart.

The identified proteins were divided into four different categories, namely carbon metabolism (six proteins), amino acid and protein biosynthesis (seven proteins), nucleotide biosynthesis and metabolism (three proteins), and antioxidant processes (two proteins) (Figure 2B). Proteins involved in carbon metabolism included glycolytic enzymes (FBA1, TDH3, GPM1, and ENO2) as well as enzymes involved in the conversion of pyruvate to ethanol (PDC1, ADH1) (Figure 2A). Proteins FBA1, GPM1, ENO2, and PDC1 were downregulated whereas TDH3 upregulated in the Δ*sod1* mutant (Figure 1 and Figure 2). In turn, the level of ADH1 protein was the same for both tested strains (Figure 1 and Figure 2). Within the next group of the identified proteins, there were those related to the biosynthesis of amino acids (MET6, LEU2, HIS7, and DYS1) and proteins (EFT1/EFT2, TEF1/TEF2, and RPP2B) (Figure 2A). The proteins MET6, LEU2, HIS7, DYS1, EFT1/EFT2, and RPP2B were downregulated and TEF1/TEF2 were upregulated in the Δ*sod1* mutant (Figure 1 and Figure 2). Another group contained the proteins participating in nucleotide biosynthesis and metabolism, ADE17/ADE16, ADO1, and GUK1. These proteins were downregulated in the Δ*sod1* mutant (Figure 1 and Figure 2). Moreover, two antioxidant proteins, TSA1 and AHP1, were detected and showed increased expression (Figure 1 and Figure 2).

### 3.4. Decrease of Methionine Synthase Expression Causes Methionine Auxotrophy in the Δsod1 Mutant

**Figure 3:**
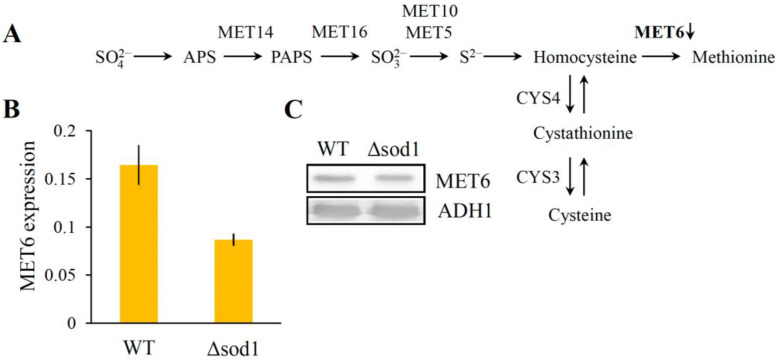
The level of methionine synthase (MET6) in the WT strain and Δ*sod1* mutant. (**A**) Biosynthesis of sulfur amino acids. APS—5′-adenylylsulfate; PAPS—3′-phospho-5′-adenylylsulfate; MET14—APS kinase; MET16—PAPS reductase; MET10—α-subunit of sulfite reductase complex; MET5—β-subunit of sulfite reductase; CYS4—cystathionine β-synthase; CYS3—cystathionine γ-lyase; downward arrow indicates protein downregulation in the Δ*sod1* mutant compared to the WT strain. (**B**) The expression of the *MET6* gene and (**C**) the content of MET6 protein in the WT strain and the Δ*sod1* mutant.

The methionine synthase (MET6) is a cobalamin-independent methionine synthase involved in methionine biosynthesis and catalyzes the conversion of homocysteine to methionine (Figure 3A). The proteome analysis clearly shows that the accumulation of the MET6 protein was downregulated in the Δ*sod1* mutant (spot number two of Figure 1A). In order to determine the MET6 protein content, the western blot method with anti-MET6 antibodies was also employed. These results confirmed proteome analysis findings, showing a lower content of this protein in the Δ*sod1* mutant (Figure 3C and Figure S2). Moreover, the level of *MET6* gene expression using the −ΔΔC_T_ method for comparison to the WT strain and the Δ*sod1* mutant was calculated. It was shown that the expression of the *MET6* gene was downregulated (1.9 times lower) in the Δ*sod1* mutant (Figure 3B). These results demonstrate that the decreased expression of the *MET6* gene and the content of the MET6 protein may lead to methionine auxotrophy in the Δ*sod1* mutant.

## 4. Discussion

The role of superoxide dismutase in oxidative stress protection is well understood and has been thoroughly studied since the discovery of this enzyme [40]. The yeast *Saccharomyces cerevisiae* is a useful model for this research. The yeast strain lacking Sod1p was described for the first time by Bilinski et al. [15]. Depletion of *SOD1* caused an increase in the level of intracellular superoxide and secondary ROS that were found to react with proteins, lipids, and nucleic acids. Almost 70% of all oxidized molecules in oxidatively stressed cells are of proteinaceous nature, indicating that proteins are the most prominent in vivo targets of oxidants. Protein oxidation has often been associated with the functional decline of proteins [41,42]. Therefore, in order to explain the pleiotropic effects of *SOD1* deficiency in the yeast cells, we decided to compare the proteomes of the WT strain and the Δ*sod1* mutant employing two-dimensional gel electrophoresis. It should be noted here that previously, O’Brien et al. [24] showed the 2-DE results of mitochondrial proteins expression in the WT, Δ*sod1*, Δ*sod2*, and Δ*sod1*Δ*sod2* mutants, as well as in the WT and Δ*sod1* mutant cells treated with paraquat. The authors documented that most of the protein spots had the same level of expression in both the Δ*sod1* mutant and the wild-type strain. Only for Idh2p, Ilv5p, Ilv2p, and Aco1p was the abundance of proteins was decreased [24]. According to our knowledge, the whole cellular proteomes of the WT yeast and the strains deficient in *SOD1* have not been compared in such form yet. In our study, we used whole-cell protein extracts, which contain mainly cytosolic proteins. Eighteen proteins were observed, whose expression was altered in the Δ*sod1* mutant as compared to the wild-type strain (Figure 1). These proteins were found to be involved in carbon metabolism, amino acid and protein biosynthesis, nucleotide biosynthesis, and metabolism as well as antioxidant processes (Figure 2).

The glycolytic pathway plays a fundamental role in providing metabolic energy and intermediates during the fermentative growth of the yeast *S. cerevisiae*. Glycolytic enzymes such as FBA1, TDH3, GPM1, and ENO2 as well as PDC1, which is a key enzyme in alcoholic fermentation, were shown to have different expression levels in the Δ*sod1* mutant compared to the wild-type strain (Figure 1 and Figure 2). The decrease of expression of FBA1, GMP1, ENO2, and PDC1 proteins may account for the lower growth rate and altered sensitivity to various factors as earlier observed for the Δ*sod1* mutant [21,23,34,43]. The results of this study confirm the observations of Sehati et al. [44] relating to the metabolic alterations in the Δ*sod1* mutant during growth on glucose-supplemented media. The authors suggest that the increased level of superoxide anion either interferes with the cell signaling by redox-active molecules or damages key cellular components [44]. Interestingly, the level of TDH3 protein in the mutant lacking *SOD1* was elevated significantly (spot number 13 of Figure 1A,B). The *TDH1*, *TDH2*, and *TDH3* genes encode glyceraldehyde-3-phosphate dehydrogenase isozymes whose activity is required for both glycolysis and gluconeogenesis. The proteins Tdh2p and Tdh3p are typically present in exponentially growing cells whereas Tdh1p is primarily detected during the stationary phase [45]. As our extracts were prepared from yeast harvested at the exponential phase of growth, the above observations can explain the presence of only one of three isozymes in our model (Figure 1, Table 1). In addition to its well established metabolic function, Tdh3p has been shown to participate in several nonmetabolic processes, including DNA repair, tRNA export, regulation of mRNA stability, membrane fusion and transport, cytoskeletal dynamics, and the initiation of apoptosis [46]. Moreover, Tdh3p may interact with the NAD^+^-dependent histone deacetylase (Sir2) in the nucleus and promote Sir2-dependent gene silencing [47]. Overexpression of the *TDH3* gene results in a decrease in the growth rate and extension of a G1 phase of the cell cycle [48]. The induction of TDH3 protein, as shown in this study (Figure 1 and Figure 2), may be one of the reasons for significant prolongation of the G1 cell cycle phase in the Δ*sod1* mutant, as compared to the wild-type strain (i.e., 89 min for the Δ*sod1* mutant and 42 min for the WT strain, both in the DBY747 genetic background; [49]). In turn, the extension of the G1 phase may lead to a prolonged generation time, which in the case of Δ*sod1* mutant was approximately 36% longer (i.e., 121.3 ± 4.67 min for the Δ*sod1* mutant and 89.2 ± 1.87 min for the WT strain, both in SP4 genetic background; [32]). Note that this effect was also observed for different genetic backgrounds [19,32,43,49].

The Δ*sod1* mutant exhibits methionine and lysine auxotrophies when grown in air, though not when grown anaerobically [15]. Earlier studies on the origin of the methionine auxotrophy indicate that this effect may result from the O_2_-dependent toxicity of sulfite (SO_3_^2−^), an intermediate occurring upon normal reductive assimilation of sulfate by yeast [50]. Here, we show that the MET6 protein in the Δ*sod1* mutant was downregulated (spot number two of Figure 1A,B). The MET6 is a cobalamin-independent methionine synthase localized in the cytosol [51] which is involved in methionine biosynthesis (Figure 3A). Mutants that lack the *MET6* gene are methionine auxotrophs [52]. Our results indicate that methionine auxotrophy in the Δ*sod1* mutant may not only be associated with the O_2_-dependent toxicity of sulfite, but also with the decreased content of the MET6 protein (Figure 1, Figure 3C and Figure S2), which is a consequence of decreased expression of the *MET6* gene (Figure 3B). This hypothesis is supported by the fact that the Δ*sod1* mutant does not need cysteine supplementation to enable it to grow on a minimal medium since cys are formed upon the pathway, the same as that for methionine synthesis (note that MET6 is the only enzyme that differentiates these two pathways; Figure 3A, [52]). On the other hand, no changes were observed in the Δ*sod1* mutant as studied for the expression of proteins related to the synthesis of lysine (Figure 1). The latter fact can be explained by the different metabolic backgrounds of lysine and methionine auxotrophies. Lysine auxotrophy can be attributed to the superoxide inactivation of Lys4p, a homoaconitase catalyzing the conversion of homocitrate to homoisocitrate, which is the second step in the lysine biosynthesis pathway. Sod1p protects Lys4p against oxidation by reducing the level of the superoxide anion [17]. Therefore, we could observe a Lys auxotrophic phenotype in the Δ*sod1* mutant, although the expression of the Lys-relevant proteins remained unchanged. Moreover, we demonstrated a decreased expression of the LEU2 protein in the Δ*sod1* mutant (spot number nine of Figure 1A,B). LEU2 is a beta-isopropylmalate dehydrogenase that catalyzes the third step in leucine biosynthesis. Leucine auxotrophy for both studied strains (WT and Δ*sod1*) is related to genetic background (SP4 MATα *leu1 arg4*; [33]). Lack of LEU1, the isopropylmalate isomerase that catalyzes the second step in the leucine biosynthesis, causes the repression of this pathway. Other enzymes involved in Leu biosynthesis, including LEU2, are therefore no longer required and may be present in a reduced amount. For that reason, the observed changes in the expression of the LEU2 protein (Figure 1A,B) do not lead to further changes in the Δ*sod1* mutant phenotype.

Another group of proteins with altered expression in the Δ*sod1* mutant were the ones related to the biosynthesis and metabolism of purine nucleotides. Purines are vital for cell function and cell proliferation through their effects on DNA, RNA, and ATP synthesis. ADO1 is an adenosine kinase required for the utilization of S-adenosylmethionine. This enzyme is also involved in the recycling of adenosine produced through the methyl cycle by converting it to adenosine monophosphate (AMP). The yeast strain lacking the *ADO1* gene showed a reduced growth rate and revealed an extended doubling time of approximately 30% when grown in a rich medium [53]. In the present work, we provide evidence that the ADO1 protein in the Δ*sod1* mutant is downregulated (spot number 12 of Figure 1A,B). This is yet another factor that, in addition to the changes in carbon metabolism, may account for the Δ*sod1* mutant reduced growth rate and prolonged generation time. The Δ*sod1* mutant was also shown to accumulate less HIS7 protein relative to the WT strain (spot number five of Figure 1A,B). HIS7 is an imidazole glycerol-phosphate synthase, also called glutamine amidotransferase:cyclase, which catalyzes the fifth step in histidine biosynthesis [54]. Both the His and purine biosynthetic pathways are connected through the 5′-phosphoribosyl-5-amino-4-imidazole carboxamide (AICAR) cycle. AICAR, a byproduct of His biosynthesis, is also a purine precursor [54]. Mutants that lacked the *HIS7* gene were histidine auxotrophs [55]; however, a decreased expression of His7p, as observed in our study for the Δ*sod1* mutant does not result in histidine auxotrophy but may account for the disturbance of purine synthesis. Here, it should be noted that the genes *ADE17* and *ADE16* encode AICAR transformylase isoenzymes that catalyze the penultimate step of the de novo purine biosynthesis pathway. These enzymes are bifunctional and can also produce an inosine monophosphate (IMP) cyclohydrolase activity. Ade17p is the dominant isoenzyme and appears to be an adenine-responsive enzyme, strongly repressed by adenine, whereas Ade16p is the minor isoenzyme, unresponsive to adenine levels. A disruption of the two genes resulted in adenine auxotrophy, while the expression of either gene alone was sufficient to support growth in the absence of adenine [56,57]. In this study, we have found that ADE17/ADE16 proteins were downregulated in the Δ*sod1* mutant (spot number three of Figure 1A,B). The lowered expression of these proteins was earlier shown to limit purine biosynthesis which, in turn, may result in a significant decrease in a relative lifespan (change in relative viability as a function of time in a stationary phase) [58]. It is known that the chronological and replicative lifespans of the Δ*sod1* mutant are shortened dramatically compared to the wild-type strain [29,30,31,32], and a reduced rate of purine biosynthesis may be one of the reasons for the above phenomenon. The results of this study support such an explanation since we demonstrated that the Δ*sod1* mutant had a decreased expression of a GUK1 protein (spot number 16 of Figure 1A,B). GUK1 is a guanylate kinase that converts guanosine monophosphate (GMP) to guanosine diphosphate (GDP). As shown earlier, the lack of the *GUK1* gene led to GMP accumulation and, by a feedback mechanism, it inhibited hypoxanthine-guanine phosphoribosyltransferase activity (adenine derepression process; [59]). Therefore, the reduced expression of Guk1p may be another cause of the lower growth rate of the Δ*sod1* mutant when compared to the WT strain.

Changes in carbon metabolism and the deregulation of purine biosynthesis have a significant impact on the functioning of Δ*sod1* mutant cells, including protein biosynthesis. The altered expression of proteins involved in this process was also observed in this study (Figure 1 and Figure 2). The translation initiation factor 5A (eIF5A) is encoded by *TIF51A* and *TIF51B* paralogous genes at aerobic conditions. It is the only protein that contains an essential amino acid hypusine, formed by a posttranslational modification of a specific lysine (residue 51 in *S. cerevisiae*). Hypusination is a two-step process catalyzed consecutively by deoxyhypusine synthase (Dys1p) and deoxyhypusine hydroxylase, and it is absolutely required for protein function [60,61]. The *DYS1* gene is essential for cell viability in yeast [62]. Here, we show that in the Δ*sod1*, the DYS1 protein was downregulated (spot number eight of Figure 1A,B), which might decrease the hypusination of eIF5A. As documented earlier, a depletion of eIF5A in yeast resulted in a hampered protein synthesis and led to an increase in a number of G1-arrested cells [60]. Also, a significant prolongation of the Δ*sod1* mutant G1 phase of the cell cycle was observed [49].

In yeast, ribosomes have lateral protuberances called stalks. The stalk consists of five P proteins, P0, with a molecular mass of 34 kDa, and four small acidic proteins of 11 kDa, P1A, P1B, P2A, and P2B, all capable of forming a pentameric complex P0-(P1A-P2B)/(P1B-P2A). This structure binds to a region of 26S rRNA termed a GTPase-associated domain and plays a crucial role in protein synthesis. A P1A-P2B protein complex is a key element in stalk formation, possessing structural and functional importance, whereas the P1B-P2A protein complex is implicated in the regulation of stalk function [63]. P1/P2 heterodimers bind to P0 when both ribosomal subunits are joined and committed to translation, and they detach from the stalk just after the small and large ribosomal subunits separate from the mRNA [64]. Our study reveals downregulation of the RPP2B protein in the Δ*sod1* mutant (spot number 19 of Figure 1A,B), which may hinder the formation of the described protein complexes. Ribosomal translation activity regulation is based on the reversible phosphorylation of P proteins. One of the enzymes responsible for this reaction is protein kinase 60S (PK60S). Sod1p can directly influence the PK60S activity [65,66]. A mechanism of this regulation is connected with the content of amino acid clusters present in a SOD protein, similar to those occurring in the P1/P2 proteins and recognized by PK60S (competitive inhibition, [66,67]). In the case of the Δ*sod1* mutant, a change in P-protein phosphorylation may have a regulatory effect on the rate and function of the cell translational system, and in consequence, result in an extended time of generation and altered cell size [68,69]. Our earlier results showed that cell size in the population of the Δ*sod1* mutant is significantly higher than WT cells by about 5% and is in the range of 4.0–6.8 µm, whereas the WT cells take size in the range of 3.6–6.0 µm [23]. What is more, cells devoid of Sod1p show a higher rate of increase in size per generation [69]. These differences between the Δ*sod1* mutant and the wild-type strain were even more apparent in the case of cells arrested in the cell division phase (pheromone α-treated cells, [68]).

The ribosomal stalk interacts with the elongation factor two (EF2) which is encoded by *EFT1* and *EFT2* paralogous genes. EF2 catalyzes the translocation of the ribosome along the messenger RNA, presumably by stimulating ribosomal gross rearrangement that results in peptidyl-tRNA transfer and the translocation of mRNA by one codon [70,71]. Justice et al. [70] demonstrated that the use of sordatin, which acts as a selective inhibitor of fungal EF2, resulted in the inhibition of protein synthesis and led to cell death. In this work, the EFT1/EFT2 proteins were downregulated in the case of Δ*sod1* mutant (spot number one of Figure 1A,B), which might possibly slow down the protein synthesis and thus indirectly explain the longer time required for cell division [19,32,49]. On the other hand, the Δ*sod1* mutant revealed an enhanced accumulation of translational elongation factor one alpha (eEF1A) (spot number one of Figure 1A,B) which is encoded by *TEF1* and *TEF2* paralogous genes. eEF1A and EF2 are members of the GTPase superfamily of proteins. The eEF1A activity is stimulated by the binding of aminoacyl-tRNA, ribosomes, and most importantly, by the presence of a codon–anticodon match between the aminoacyl-tRNA and the A site codon of the ribosome-bound mRNA. The regulation of the eEF1A activity by GTPase-activating proteins and guanine-nucleotide exchange factors is important for efficient and accurate protein synthesis and, in consequence, for cell growth [72]. Interestingly, the overexpression of *TEF1* or *TEF2* was shown to increase cell size, interfere with the cell cycle, and slow down the yeast growth [73]. All of the above effects have been observed for the case of the *SOD1*-deficient mutant and were contrasted with the WT strain [32,69].

Surprisingly, among the entire group of antioxidant proteins, in the Δ*sod1* mutant, induction of expression was observed only for the proteins TSA1 and AHP1 (spots numbered 15 and 17 of Figure 1A, respectively; Figure 1B). Tsa1p (a thioredoxin peroxidase) and Ahp1p (a thiol-specific peroxiredoxin) are members of the peroxiredoxin family within which they have the highest and the second-highest expression levels, respectively. Peroxiredoxins (Prx) are ubiquitous thiol-specific proteins, which take part in many cellular processes. Primarily, Prx reduces hydrogen peroxide and other peroxide substrates using electrons from NADPH in the thioredoxin-dependent redox system of thiol-sulfide exchange between catalytic cysteines [74]. Moreover, Tsa1p can also act as a molecular chaperone that binds the unfolded proteins and prevents their aggregation [75,76]. The elevated expression of TSA1 and AHP1 in the Δ*sod1* mutant as compared to the WT strain is seemingly a cell response to ROS. The time-dependent changes in the expression of Tsa1p under oxidative stress, which could be caused by changes in protein localization, stability, translation, and by posttranslational modification, were shown previously [77]. Therefore, apart from the increased level of glutathione, the elevated levels of TSA1 and APH1 seem to contribute to the compensative reaction of Δ*sod1* cells, providing partial protection against oxidative stress resulting from the increased superoxide level.

The pleiotropic effects of the CuZnSOD deficiency can be conditioned on several levels, including changes in the proteome and altered regulation of activities of cellular proteins; both can result in alterations of the levels of intracellular metabolites. Our study demonstrated that many of these effects can be ascribed to the changes in the protein profile. For this reason, protein profile analysis from one side indicates the already established role of Sod1p in oxidative stress protection and from the other side suggests its less obvious role in the signaling pathways, as was recently postulated [10]. The obtained results may shed new light on the understanding of the phenotype effects resulting from Sod1p dysfunction observed in many human diseases including neurodegenerative diseases, cancer, and age-related diseases.

## 5. Conclusions

This study provides comparative proteome mapping of the wild-type strain and the SOD1-deficient mutant, focusing especially on the Sod1p-affected proteins in the yeast *S. cerevisiae*. Such an approach offers a global overview of oxidative changes elicited by the disruption of this important antioxidant enzyme. We carried out a systematic quantitative analysis of alterations detected in proteomic 2-DE maps followed by an MS-based protein identification for each differentiating spot. The results bring an explanation to some effects of *SOD1* depletion in the yeast such as changes in the carbon metabolism, methionine auxotrophy, prolongation of the G1 phase of the cell cycle, extended time of generation, shortened lifespan, changes in cell size, as well as altered level of protein synthesis. Completion of these data by metabolomic analysis should allow for a better understanding of metabolic alterations caused by the lack of CuZnSOD, and thus, the biological role of this crucial antioxidant enzyme.

## Figures and Tables

**Table 1:** List of proteins identified by mass spectrometry analysis.

Spot	Accession Number (ORF)	Protein Name	Description	Molecular Mass [kDa]		pI ^b^	MOWSE Score	Number of Peptides Matched	Sequence Coverage [%]	emPAI
				Calculated ^a^	Theoretical ^b^					
1.	YOR133W YDR385W	EFT1 EFT2 ^p^	Elongation factor 2 Elongation factor 2	93.69 93.69	93.28 93.28	6.23 6.23	3559 3559	72 72	39 39	5.85 5.85
2.	YER091C	MET6	Cobalamin-independent methionine synthase	85.98	85.85	6.41	3703	64	35	5.02
3.	YMR120C YLR028C	ADE17 ADE16 ^p^	Enzyme of ‘de novo’ purine biosynthesis Enzyme of ‘de novo’ purine biosynthesis	65.56 65.64	65.26 65.28	6.52 6.52	2280 1075	41 18	26 14	4.02 1.19
4.	YLR044C	PDC1	Major of three pyruvate decarboxylase isoenzymes	61.68	61.49	6.11	2856	54	19	4.54
5.	YBR248C	HIS7	Imidazole glycerol phosphate synthase	61.54	61.05	5.15	1219	22	18	1.83
6.	YPR080W YBR118W	TEF1 TEF2 ^p^	Translational elongation factor 1 alpha Translational elongation factor 1 alpha	50.40 50.40	50.04 50.04	9.58 9.58	684 684	16 16	10 10	1.01 1.01
7.	YHR174W	ENO2	Enolase II, a phosphopyruvate hydratase	46.94	46.91	5.85	9716	150	35	43.84
8.	YHR068W	DYS1	Deoxyhypusine synthase	43.26	42.88	5.49	643	11	9	1.09
9.	YCL018W	LEU2	Beta-isopropylmalate dehydrogenase	39.04	38.95	5.48	872	16	15	2.67
10.	YOL086C	ADH1	Alcohol dehydrogenase I	37.28	36.84	6.67	4286	89	20	9.83
11.	YKL060C	FBA1	Fructose 1,6-bisphosphate aldolase	39.88	39.61	5.59	1043	18	11	2.05
12.	YJR105W	ADO1	Adenosine kinase	36.52	36.36	4.77	1047	15	10	1.84
13.	YGR192C	TDH3	Glyceraldehyde-3-phosphate dehydrogenase	35.84	35.74	6.96	1978	39	21	7.35
14.	YKL152C	GPM1	Tetrameric phosphoglycerate mutase	27.59	27.61	9.34	706	13	10	2.92
15.	YML028W	TSA1	Thioredoxin peroxidase	21.69	21.58	4.77	957	20	9	3.88
16.	YDR454C	GUK1	Guanylate kinase	20.68	20.64	7.34	789	15	7	2.88
17.	YLR109W	AHP1	Thiol-specific peroxiredoxin	19.27	19.11	4.78	831	13	9	3.99
18.	YJR104C	SOD1	Cytosolic copper-zinc superoxide dismutase	15.96	15.85	5.91	963	16	10	9.06
19.	YDR382W	RPP2B	Ribosomal protein P2 beta	11.04	11.03	3.80	793	11	3	1.27

^a^ Molecular mass as calculated by mass spectrometry analysis, ^b^ Molecular mass and pI as given in the *Saccharomyces* Genome Database, ^p^ Paralogous proteins.

## Data Availability

The data presented in this study are available on request from the corresponding author. The data are not publicly available due to privacy.

## Supplementary Materials

The following supporting information can be downloaded at: https://www.mdpi.com/article/10.3390/metabo13030459/s1, Figure S1: Comparison of proteomes of the wild-type (WT) strain and the Δ*sod1* mutant which was performed by two-dimensional gel electrophoresis (2-DE) of whole-cell protein extracts; Figure S2: The methionine synthase (MET6) and alcohol dehydrogenase (ADH1) content in the wild-type (WT) strain and the Δ*sod1* mutant.
