# Supplementary material for: Changes in a Protein Profile Can Account for the Altered Phenotype of the Yeast Saccharomyces cerevisiae Mutant Lacking the Copper-Zinc Superoxide Dismutase
Source: Metabolites. 2023 Mar 22;13(3):459. doi: 10.3390/metabo13030459 (PMC10056615; doi:10.3390/metabo13030459)
Supplement: Supplementary file 1 [file metabolites-13-00459-s001.zip › metabolites-2264884-supplementary.pdf]

Changes in a Protein Profile Can Account for the Altered Phenotype of the Yeast Saccharomyces cerevisiae Mutant Lacking the Copper-Zinc Superoxide Dismutase

Magdalena Kwolek-Mirek 1,* , Aleksandra Dubicka-Lisowska 2, Sabina Bednarska 1, Renata Zadrag-Tecza 1,* and Pawel Kaszycki 2

1 Department of Biology, Institute of Biology and Biotechnology, College of Natural Sciences, University of Rzeszow, 35-601 Rzeszow, Poland; sbednarska@ur.edu.pl
2 Department of Plant Biology and Biotechnology, Faculty of Biotechnology and Horticulture, University of Agriculture in Krakow, 31-425 Krakow, Poland; pawel.kaszycki@urk.edu.pl (P.K.)
* Correspondence: mkwolek@ur.edu.pl (M.K.-M.); retecza@ur.edu.pl (R.Z.-T.); Tel.: +48-17-785-5412 (M.K.-M.); +48-17-785-5413 (R.Z.-T.)

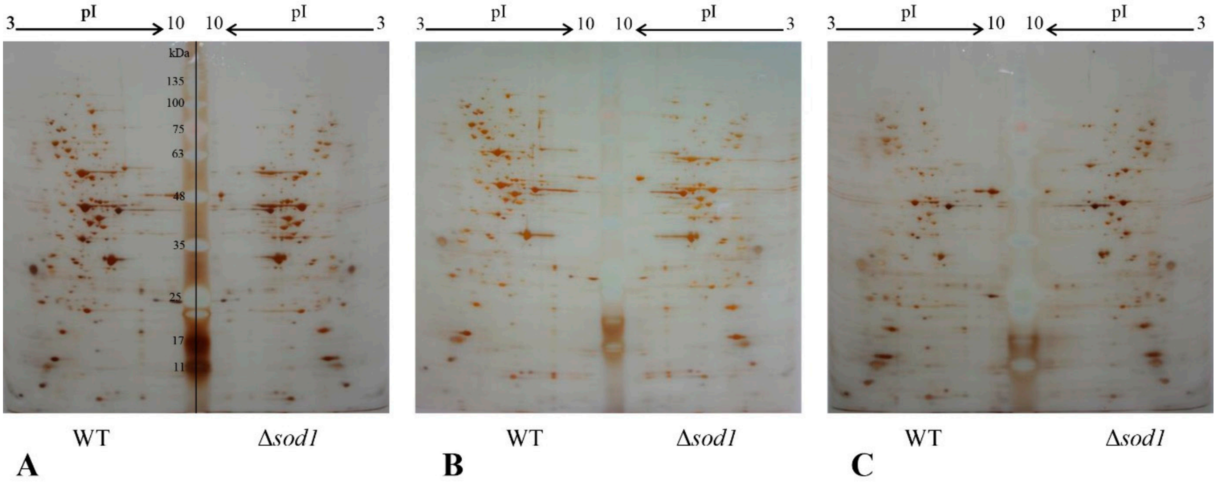

Figure S1. Comparison of proteomes of the wild-type (WT) strain and the Δ*sod1* mutant which was performed by two-dimensional gel electrophoresis (2-DE) of whole-cell protein extracts. In the isoelectrofocusing step protein samples were loaded onto 7 cm IPG strips with pI ranging from 3 to 10. The SDS-PAGE step was carried out using Protean II xi Cell slab unit. Proteins were detected with silver staining. (A-C) For proteome mapping and matching three protein extracts (both the WT and Δ*sod1*) obtained upon three independent experiments were used. The results are presented as original images of gels.

See also Figure 1

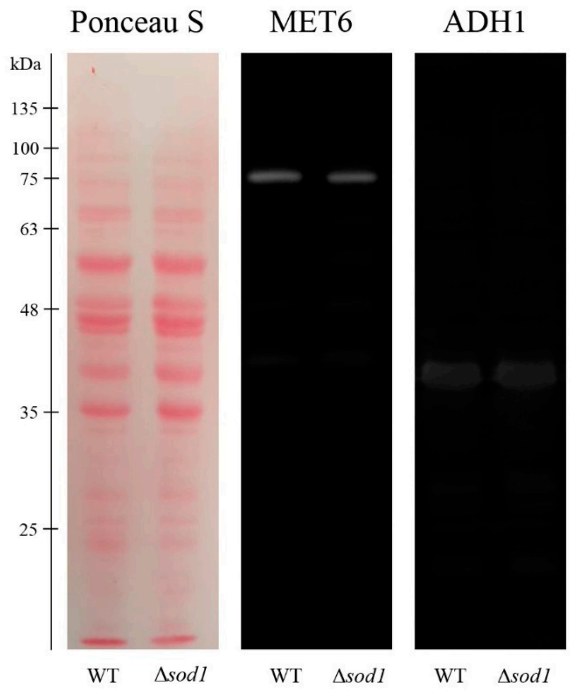

Figure S2. The methionine synthase (MET6) and alcohol dehydrogenase (ADH1) content in the wild-type (WT) strain and the Δ*sod1* mutant which was determined by immunoblotting method using anti-yeast methionine synthase (1:500, X-P05694-N, Abmart) and anti-yeast alcohol dehydrogenase (1:5000, ab34680, Abcam) antibodies. The presence of proteins on the membrane was confirmed by Ponceau S. The results are presented as original images of blots.

See also Figure 3
